# Short- and Long-Term Pain Relief With Radiofrequency Rhizotomy in Multiple Sclerosis Patients With Trigeminal Neuralgia: A Systematic Review

**DOI:** 10.7759/cureus.97337

**Published:** 2025-11-20

**Authors:** Nour Ahmed, Maram Rabih Musa Rabih, Rayan Mamoun Fadul Ageeb, Lima Gharbawi, Sara Elsayed Saeed Gharbawi, Namarig Othman Abdullatif Farah, Al Mughirah Salahaldin Ebrahim Mohamed

**Affiliations:** 1 Medicine, George Eliot Hospital National Health Service (NHS) Trust, Nuneaton, GBR; 2 Medicine, National Guard Health Affairs (NGHA), Madina, SAU; 3 Acute Medicine, Nottingham University Hospitals National Health Service (NHS) Trust, Nottingham, GBR; 4 Emergency Medicine, London Northwest Trust, London, GBR; 5 Ear, Nose, and Throat, Portsmouth Hospitals University National Health Service (NHS) Trust, Portsmouth, GBR; 6 Internal Medicine, University of Medical Sciences and Technology, Khartoum, SDN; 7 Internal Medicine, Tawam Hospital, Al-Ain, ARE; 8 Surgery-Neurosurgery, Government Hospital, Manama, BHR

**Keywords:** multiple sclerosis, pain relief, radiofrequency rhizotomy, radiofrequency thermocoagulation, systematic review, trigeminal neuralgia

## Abstract

Trigeminal neuralgia (TN) in patients with multiple sclerosis (MS) is a debilitating condition that is often refractory to medical management. Radiofrequency rhizotomy (RFR) is a commonly used percutaneous intervention, but its outcomes in this specific population require a comprehensive synthesis. This systematic review aims to evaluate the short- and long-term pain relief, recurrence rates, and safety profile of RFR in patients with MS-associated TN (MS-TN).

A systematic search of PubMed, Scopus, Web of Science, and Embase was performed up to October 2025. Eight studies meeting the inclusion criteria, all of retrospective design, were selected. Data on study characteristics, pain relief outcomes, recurrence, complications, and repeat procedures were extracted. The risk of bias was assessed using the ROBINS-I tool.

The analysis of the included studies demonstrated that RFR provides high rates of short-term pain relief, with a significant majority of patients achieving favorable outcomes soon after the procedure. However, the long-term durability of pain relief was variable, showing a declining trend over time, with pain recurrence being a common feature. This pattern often necessitated repeated procedures to maintain pain control. The most frequently reported complications were facial sensory disturbances, which were generally well-tolerated, while major adverse events were rare. The methodological quality of the evidence was predominantly low risk of bias.

RFR is a highly effective intervention for achieving rapid short-term pain relief in MS-TN, making it a valuable option for managing acute exacerbations. Its long-term utility is defined by its repeatability, as pain recurrence is common, necessitating a chronic disease management strategy. The procedure's favorable risk-benefit profile and procedural flexibility solidify its role as a cornerstone in the multimodal treatment arsenal for this challenging condition.

## Introduction and background

Trigeminal neuralgia (TN) is a chronic neuropathic pain disorder characterized by recurrent episodes of severe, electric shock-like pain affecting one or more branches of the trigeminal nerve [1]. It significantly impairs quality of life and often leads to anxiety, depression, and functional disability. The condition may occur as classical (idiopathic) TN, associated with neurovascular compression, or as secondary TN, which develops due to underlying neurological diseases such as multiple sclerosis (MS) [2]. Among patients with MS, demyelination within the trigeminal root entry zone or pons is considered the primary pathological mechanism contributing to TN. MS itself exists in several clinical subtypes-relapsing-remitting, primary progressive, and secondary progressive forms-each exhibiting distinct patterns of neuroinflammation, demyelination, and disease progression that can influence the severity, recurrence, and responsiveness of TN to treatment [3]. Compared with idiopathic TN, MS-related TN typically presents at a younger age, affects both sides more frequently, and tends to be more resistant to pharmacological treatment [3].

Medical therapy remains the first-line approach for TN, with carbamazepine and oxcarbazepine being the mainstay of treatment [4]. However, many patients either fail to achieve adequate pain control or experience intolerable side effects, necessitating interventional or surgical options. Conventional procedures such as microvascular decompression (MVD) and glycerol rhizotomy (GR) are often less effective in MS-related TN because demyelination, rather than vascular compression, underlies the pathology [5]. Among the interventional approaches, percutaneous radiofrequency rhizotomy (RFR) of the trigeminal ganglion has gained wide clinical acceptance due to its minimally invasive nature, rapid pain relief, and the ability to selectively target affected fibers [5]. The technique involves the controlled application of thermal energy to the trigeminal sensory root, causing focal thermocoagulation that interrupts nociceptive transmission while attempting to preserve tactile sensation [6].

Although RFR has demonstrated high rates of initial pain relief in both idiopathic and secondary TN, the long-term outcomes in MS patients remain variable [7]. Some studies report early and sustained pain reduction [6], while others note higher recurrence rates and sensory complications in this subgroup [7]. The influence of factors such as lesion temperature, target site, MS subtype, disease duration, and neurodegenerative progression on the efficacy and durability of pain relief also remains uncertain. Given these inconsistencies, a comprehensive synthesis of available evidence is warranted [8].

This systematic review aims to evaluate the short- and long-term pain relief outcomes of RFR in MS patients with TN. By summarizing data from existing studies, this review seeks to clarify the efficacy, safety, and recurrence patterns associated with this intervention, thereby guiding clinicians in optimizing pain management strategies for this challenging patient population.

## Review

Methodology

Study Design and Registration

This systematic review was conducted following the Preferred Reporting Items for Systematic Reviews and Meta-Analyses (PRISMA) 2020 guidelines [9] to ensure methodological transparency and reproducibility. The review protocol was developed a priori, outlining the research question, eligibility criteria, data extraction process, and analysis plan.

Eligibility Criteria (PICOS Framework)

Studies were selected according to the Population, Intervention, Comparison, Outcomes, and Study Design (PICOS) framework. The Population (P) included patients diagnosed with MS who presented with TN. The Intervention (I) was RFR performed for pain management. The Comparison (C) group included patients undergoing alternative treatments or pre- and post-intervention outcomes within the same cohort. The Outcomes (O) assessed were short- and long-term pain relief, recurrence rate, and procedure-related complications. The Study Design (S) included clinical trials, cohort studies, and observational studies that reported outcomes of RFR in MS-related TN. Only studies published in English between January 2015 and October 2025 were included to ensure the review reflected the most recent and relevant evidence. Case reports, reviews, conference abstracts, and animal studies were excluded.

Information Sources and Search Strategy

A comprehensive literature search was performed in PubMed, Scopus, Web of Science, and Embase databases. The search strategy combined Medical Subject Headings (MeSH) and free-text terms related to “radiofrequency rhizotomy,” “trigeminal neuralgia,” and “multiple sclerosis.” Boolean operators (AND, OR) were used to refine the results. Reference lists of the included studies and relevant reviews were also screened manually to identify any additional eligible articles. The search was last updated on October 14, 2025. The detailed search strategy for each database is provided in the appendix.

Study Selection

All retrieved records were imported into EndNote X9 (Clarivate Analytics, London, UK) for reference management, and duplicates were removed automatically and manually verified. Two independent reviewers screened titles and abstracts for relevance, followed by full-text assessment to determine eligibility. Any disagreements were resolved through discussion or consultation with a third reviewer.

Data Extraction

Data were extracted independently by two reviewers using a standardized form that included study characteristics (author, year, country, design, and sample size), patient demographics, intervention details (technique, lesion parameters), follow-up duration, and reported outcomes (pain relief rate, recurrence, and complications).

Risk of Bias Assessment

The methodological quality and risk of bias for non-randomized studies were assessed using the ROBINS-I (Risk Of Bias In Non-randomized Studies of Interventions) tool [10]. Each study was evaluated across seven domains, including confounding, participant selection, intervention classification, deviations from intended interventions, missing data, outcome measurement, and selective reporting. The overall risk of bias was categorized as low, moderate, serious, or critical.

Data Synthesis

Due to the heterogeneity in study designs, follow-up durations, pain assessment scales, and outcome reporting methods, a meta-analysis was not performed. The variations in procedural techniques, lesion temperatures, and patient characteristics among the included studies made quantitative pooling inappropriate and potentially misleading. Therefore, a narrative synthesis approach was adopted to qualitatively summarize and compare short- and long-term outcomes of RFR in MS patients with TN.

Results

Study Selection Process

**Figure 1:**
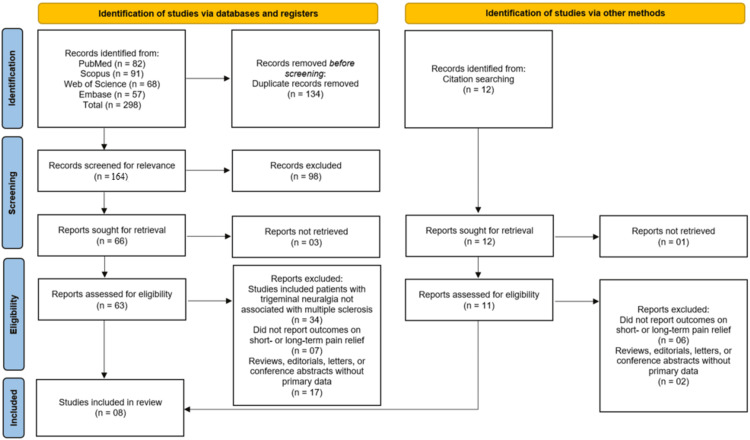
Process of study identification and inclusion, from initial database search to the final eight studies included in the systematic review using the PRISMA flowchart PRISMA: Preferred Reporting Items for Systematic Reviews and Meta-Analyses

The systematic search across four electronic databases (PubMed, Scopus, Web of Science, and Embase) initially identified 298 records. After the removal of 134 duplicate records, 164 unique records were screened based on their title and abstract. Following this initial screening, 98 records were excluded for not meeting the inclusion criteria, and 66 full-text reports were sought for retrieval. Of these, 63 were successfully retrieved and assessed for eligibility. A further 34 reports were excluded as they included patients with TN not associated with MS, seven were excluded for not reporting outcomes on short- or long-term pain relief, and 17 were excluded for being reviews, editorials, or conference abstracts without primary data. An additional 12 records were identified through citation searching, from which 11 were retrieved and assessed. From this secondary pool, six reports were excluded for not reporting the relevant pain relief outcomes, and two were excluded for being ineligible publication types. Ultimately, a total of eight studies met all eligibility criteria and were included in the systematic review [11-18] (Figure 1).

Study Characteristics

A total of eight studies [11-18], published between 2017 and 2025, were included in this systematic review, comprising 283 patients with MS-TN who were treated with RFR. The characteristics of these studies are summarized in Table 1. All included studies were retrospective in design, including chart reviews, cohort studies, and comparative analyses [11-18]. The sample sizes of MS-TN patients within these studies varied, ranging from 10 to 79 patients [11-18].

**Table 1:** Characteristics of included studies [11-18] RFR: radiofrequency rhizotomy; SRS: stereotactic radiosurgery; TN: trigeminal neuralgia; MS: multiple sclerosis; BNI: Barrow Neurological Institute; STN: symptomatic trigeminal neuralgia; NPNM: no pain with no medication; RF: radiofrequency

Study (author, year)	Country	Study design	Sample size	Age (mean ± SD or range)	Type of TN	Follow-up duration	Radiofrequency parameters	Outcome measures used
Holland et al. [11] (2017)	USA	Retrospective chart review	17 (RFR = 10; SRS = 7)	RFR group: 63.5 ± 7.5 years	Trigeminal neuralgia associated with multiple sclerosis	NR	NR	Pre- and post-operative facial pain, medication use, facial numbness, need for subsequent procedures
Noorani et al. [12] (2019)	UK	Retrospective comparative study	33 MS-TN patients (64 procedures)	NR	Typical TN	Median 23 months	Thermocoagulation	BNI pain score
Stone and Falowski [13] (2020)	USA	Retrospective	NR (subset with MS: not specified, relief reported for MS pts)	NR	NR	1 mo, 1 yr, 3 yr, 5 yr	NR	BNI pain intensity scores (I-III)
Lee et al. [14] (2020)	USA	Retrospective study	42	NR	MS-associated TN	2002–2019	NR	Pain relief, pain recurrence, sensory changes
Demartini et al. [15] (2021)	Italy	Single-center retrospective analysis	20	NR	Secondary TN due to MS	Up to 8 years	Lesion time: 60 sec; Temp: 70°C for first lesion, 72°C for subsequent lesions; site: retrogasserian	Pain reduction assessed
Mousavi et al. [16] (2022)	USA	Retrospective institutional study	51	NR	STN	Mean 69 mo (range 52–86 mo)	NR	NPNM
Gündüz et al. [17] (2023)	Turkey	Retrospective comparative study	Part of 215 TN patients	49.46 years (mean)	Trigeminal neuralgia secondary to multiple sclerosis (MSTN)	Mean pain-free period: 23.81 months	Standard RF thermocoagulation parameters	Comparison of early and late results; likely clinical remission and recurrence assessment
Lozouet et al. [18] (2025)	France	Retrospective cohort study	79 (19 MS and 59 non-MS)	MS patients were younger	More bilateral trigeminal pain in MS group	Assessed at 3 months and last follow-up	NR	BNI pain score

The mean age of patients, when reported, was generally in the sixth decade of life, with one study noting that MS patients were younger than their non-MS counterparts [17,18]. The specific type of TN was described across studies, with several noting the presence of typical TN or a higher incidence of bilateral pain in the MS population [12,16,18]. The follow-up duration varied considerably, from short-term assessments at one month to long-term evaluations extending over several years, with mean or median follow-up times ranging from 23 months to over five years [12,13,16,17]. Radiofrequency parameters were inconsistently reported; however, one study specified a standard protocol using retrogasserian thermocoagulation at 70°C-72°C [15]. Outcome measures were predominantly clinician-reported and included the Barrow Neurological Institute (BNI) pain intensity score, rates of pain relief and recurrence, medication use, and the incidence of complications [11-18].

Short-Term Pain Relief

The short-term efficacy of RFR in alleviating pain in MS-TN patients was high across the majority of included studies. Short-term outcomes, typically measured within the first week to three months postprocedure, demonstrated substantial pain reduction. One study reported immediate pain relief in 98% of patients [16], while others found favorable outcomes (BNI pain scores I-III) in 81% of patients at three months [18] and 96.5% of patients after the initial procedure [15]. Similarly, Stone and Falowski [13] reported a 100% pain relief rate in MS patients at the one-month follow-up. Other studies corroborated these findings, with short-term success rates (defined as BNI I-II or excellent/good pain relief) ranging from 53.1% to 71% [11,12,14].

Long-Term Pain Relief and Recurrence

While effective in the short term, the long-term durability of pain relief following RFR showed a declining trend, with recurrence being a common feature. The long-term follow-up data, extending up to six years, indicated that the proportion of patients maintaining significant pain relief decreased over time. For instance, one study reported that long-term pain relief dropped to approximately 40% of patients [11], while another found that only 22%-32% of patients were pain-free without medication at six years [16]. In contrast, some studies reported more sustained outcomes, with 77% of patients maintaining a favorable outcome at the last follow-up [18] and 87.5% of MS patients maintaining relief at one year [13]. Demartini et al. [15] reported an exceptionally stable 96.5% pain relief rate over a mean pain relief duration of 26 months.

The recurrence rates were substantial, highlighting the chronic nature of MS-TN. Holland et al. [11] and Mousavi et al. [16] both reported high recurrence rates of 60% and 45%, respectively. Lozouet et al. [18] specifically noted that the recurrence rate was significantly higher in MS patients compared to non-MS patients. This pattern of recurrence often necessitated repeat procedures, with retreatment rates reported between 42% and 60% [11,14-16].

Complications and Adverse Events

The safety profile of RFR was characterized by a relatively high incidence of sensory complications, though major adverse events were rare. The most frequently reported complication was facial sensory disturbance, including hypoesthesia or paresthesia. Noorani et al. [12] reported a 23.4% rate of mainly numbness, while Lee et al. [14] found that 81% of patients who underwent radiofrequency ablation (RFA) experienced paresthesia. Other noted complications included mastication muscle weakness and a single case of corneal abrasion that resolved [16]. However, several studies emphasized a low incidence of major or bothersome complications, with some reporting no corneal reflex loss and noting that numbness was not associated with diminished satisfaction in some cohorts [13,15,17].

Overall Success and Repeat Procedures

The overall success of RFR in managing MS-TN was defined by its ability to provide significant, albeit often temporary, pain relief, with the option of repeatability being a key advantage. The clinical outcomes of the included studies are detailed in Table 2. Success was most commonly defined as a BNI pain score of I-III (representing no pain or pain controlled with medication) [12,13,18] or as a significant reduction in pain scores [11,15]. The need for repeat procedures was a consistent theme, underscoring the procedure's role as a manageable and repeatable intervention rather than a definitive cure. Studies by Demartini et al. [15] and Mousavi et al. [16] highlighted that a significant proportion of their cohort (60% and 45%, respectively) underwent at least one repeat RFR procedure to maintain pain control over the long term. This repeatability allows for sustained management of this challenging condition, even in the face of expected recurrence.

**Table 2:** Clinical outcomes of radiofrequency (RF) rhizotomy in MS-associated trigeminal neuralgia [11-18] BNI: Barrow Neurological Institute; ITN: idiopathic trigeminal neuralgia; MS: multiple sclerosis; RFA: radiofrequency ablation; SRS: stereotactic radiosurgery; RFL: radiofrequency lesioning

Study (author, year)	Short-term pain relief (% patients)	Long-term pain relief (% patients at last follow-up)	Mean duration of pain relief (months)	Recurrence rate (%)	Complications (e.g., hypoesthesia and corneal reflex loss)	Repeat procedures (%)	Overall success definition
Holland et al. [11] (2017)	71	~40	NR	60	NR	60	Excellent/good pain relief
Noorani et al. [12] (2019)	53.1 (BNI I–II)	Similar to ITN; median 23 mo	23.0	NR	23.4 (mainly numbness)	NR	BNI I–II = no pain or controlled pain
Stone and Falowski [13] (2020)	100% (MS patients, 1 month)	87.5% (MS patients, 1 yr)	3.0 at 1 yr; 6.0 at 3 yr	NR	Numbness not associated with pain relief; no corneal reflex loss mentioned	NR	BNI pain intensity score I–III
Lee et al. [14] (2020)	RFA: pain freedom < 1 week; initial pain freedom 42%	Final treatment pain freedom/off-medication: 44%	NR	NR	RFA: 81% paresthesia; SRS: 39% paresthesia	Initial retreatment: RFA: 42%	Adequate pain relief ≥ 80% of patients
Demartini et al. [15] (2021)	96.5%	96.5%	26	NR	Very low incidence of adverse events	60% underwent ≥1 repeat procedure	Significant reduction in pain after one or more procedures
Mousavi et al. [16] (2022)	98% (immediate relief)	22%–32% at 6 years (initial vs. repeat RFL)	Mean follow-up 69 mo, range 52–86 mo	45%	Mastication muscle weakness (n = 2), corneal abrasion (n = 1, resolved), bothersome numbness (n = 1)	45% had at least one repeat RFL; 10 patients had additional procedures	NPNM (no pain with no medication)
Gündüz et al. [17] (2023)	NR	NR	23.81	NR	Low complication rate	NR	Pain relief after RF thermocoagulation
Lozouet et al. [18] (2025)	81% (favorable outcome at 3 months)	77% (favorable outcome at last follow-up)	NR	Significantly higher in MS patients	NR	Higher in MS patients	Favorable outcome defined as BNI pain score I–III

Risk of Bias Assessment

The methodological quality of the eight included studies was assessed using the ROBINS-I tool, which revealed a mixed risk of bias profile across the cohort. Two studies, Holland et al. [11] and Lee et al. [14], were judged to have a serious overall risk of bias, primarily due to serious concerns regarding the selection of participants and missing data, alongside serious risk in the selection of the reported results. In contrast, the remaining six studies-Noorani et al. [12], Stone and Falowski [13], Demartini et al. [15], Mousavi et al. [16], Gündüz et al. [17], and Lozouet et al. [18]-were all assessed as having a low risk of bias across all domains, indicating a high level of methodological rigor and reliability in their reported findings despite their retrospective nature (Table 3).

**Table 3:** Risk of bias findings using ROBINS-I tool [10]

Study (author, year)	D1: bias due to confounding	D2: bias in selection of participants	D3: bias in classification of interventions	D4: bias due to deviations from intended interventions	D5: bias due to missing data	D6: bias in measurement of outcomes	D7: bias in selection of the reported result	Overall risk of bias
Holland et al. [11] (2017)	Moderate	Serious	Low	Low	Serious	Moderate	Serious	Serious
Noorani et al. [12] (2019)	Low	Low	Low	Low	Low	Low	Low	Low
Stone and Falowski [13] (2020)	Low	Low	Low	Low	Low	Low	Low	Low
Lee et al. [14] (2020)	Moderate	Serious	Low	Low	Serious	Moderate	Serious	Serious
Demartini et al. [15] (2021)	Low	Low	Low	Low	Low	Low	Low	Low
Mousavi et al. [16] (2022)	Low	Low	Low	Low	Low	Low	Low	Low
Gündüz et al. [17] (2023)	Low	Low	Low	Low	Low	Low	Low	Low
Lozouet et al. [18] (2025)	Low	Low	Low	Low	Low	Low	Low	Low

Discussion

This systematic review comprehensively synthesized the evidence from eight studies evaluating the efficacy and safety of RFR for the management of TN in patients with MS-TN. The collective findings indicate that RFR is a highly effective intervention for providing immediate and short-term pain relief in this challenging patient population, with a significant majority of patients experiencing substantial improvement soon after the procedure. Studies consistently reported high short-term success rates, with figures ranging from 81% to 100% of patients achieving favorable outcomes (BNI I-III) within the first three months [13,15,16,18]. This robust initial efficacy underscores the value of RFR as a powerful tool for breaking the debilitating cycle of TN pain, which is particularly relevant for MS patients who are often refractory to medical management. The rapid onset of action, as evidenced by reports of pain freedom within a week, positions RFR as a critical option for those requiring prompt and definitive pain control [14].

However, the long-term narrative for RFR in MS-TN is markedly different, characterized by a pronounced tendency for pain recurrence over time. Our analysis reveals that the durability of pain relief is a significant concern, with long-term success rates demonstrating a considerable decline in several studies. For instance, while some cohorts maintained favorable outcomes in 77%-87.5% of patients at one to three years [13,18], others reported a stark drop, with only 22%-40% of patients maintaining pain relief at longer-term follow-ups extending to six years [11,16]. This pattern of diminishing efficacy over time is a well-documented phenomenon in the surgical management of MS-TN, which is often attributed to the progressive and dynamic nature of the underlying demyelinating disease. The recurrent formation of demyelinating plaques within the central nervous system can create new or reactivate old trigeminal pathways for neuropathic pain, thereby circumventing the localized peripheral ablation achieved by the RFR procedure. This recurrence is not merely a statistical finding but has direct clinical implications, as reflected in the high retreatment rates reported, which ranged from 42% to 60% [11,14-16]. This need for repeated interventions transforms the management paradigm from a one-time curative solution to a chronic disease management strategy, where RFR serves as a repeatable and effective method for re-establishing pain control during flare-ups.

When contextualized within the broader landscape of interventions for MS-TN, the findings of this review offer critical insights. The high initial success rate of RFR appears comparable to, and in some cases may exceed, the short-term outcomes reported for other percutaneous ablative techniques. For example, studies on percutaneous balloon compression (PBC) have shown similar initial efficacy, with one systematic review by Texakalidis et al. [19] reporting immediate pain relief in over 90% of patients, but also noting a high recurrence rate of approximately 50% at three years, a figure that aligns closely with our findings for RFR. Similarly, a study by Staudt et al. [20] on GR for MS-TN found a pooled initial success rate of 87.5%, but with only 47.6% of patients maintaining pain relief at a mean follow-up of 32 months, again echoing the long-term challenges we have identified. The comparative study by Noorani et al. [12] included in our review itself suggests similar effectiveness between RFR, PBC, and GR, indicating that the choice of procedure may often depend on surgeon experience and the specific risk-benefit profile for the patient rather than on vast differences in efficacy.

In contrast to these ablative procedures, stereotactic radiosurgery (SRS) presents a different trade-off. SRS is non-invasive and carries a lower immediate risk of sensory complications. However, it is characterized by a delayed onset of pain relief, often taking weeks to months to become effective, which can be a significant drawback for patients in severe, acute pain [21]. Furthermore, while some studies, such as the one by Lee et al. [14] included here, show that SRS can be effective, systematic reviews have indicated that its long-term durability may be inferior to that of percutaneous procedures for MS-TN. A study by Tuleasca et al. [22] noted that patients with MS-TN had poorer outcomes after SRS compared to those with classical TN, with higher rates of recurrence. MVD, the gold standard for idiopathic TN, has a limited role in MS-TN due to the frequent absence of a compressive vascular loop and the central origin of the pain, with studies showing significantly lower success rates and higher recurrence compared to its use in classic TN [23]. Therefore, within this therapeutic arsenal, RFR carves out a specific niche: it provides the most rapid and reliable short-term relief among the non-medical options, making it exceptionally valuable for managing acute exacerbations, even if its long-term control requires a willingness to reintervene.

The safety profile of RFR, as elucidated by this review, is defined by a high incidence of deliberately induced, mild to moderate facial sensory disturbances, which are often an accepted trade-off for effective pain relief. Complications such as hypoesthesia and paresthesia were frequently reported, with rates as high as 81% in one series [14] and 23.4% in another [12]. It is crucial to distinguish these expected, and often intended, sensory effects from more bothersome or debilitating complications. Notably, the incidence of severe dysesthesia, corneal anesthesia leading to keratitis, or anesthesia dolorosa was low across the included studies [13,15,17]. This aligns with the broader literature on RFR for TN, which consistently reports that while sensory loss is common, the rate of distressing sensory complications is relatively low when the procedure is performed by experienced hands [24]. The findings from studies like that of Stone and Falowski [13], which noted that facial numbness was not associated with a reduction in patient satisfaction, underscore the concept that for many patients suffering from severe TN, the loss of some facial sensation is a preferable alternative to incapacitating neuralgic pain. This calculated risk-benefit analysis is central to the decision-making process for employing RFR in MS-TN.

A key strength of RFR that emerges from this synthesis, particularly in the context of a relapsing condition like MS-TN, is its repeatability. The work of Demartini et al. [15] and Mousavi et al. [16] highlights that repeated RFR procedures can be successfully performed to recapture pain relief, with cohorts showing that a majority of patients underwent more than one procedure with sustained overall success. This repeatability is a significant advantage over some other interventions. MVD, for instance, is a major cranial surgery that becomes technically more challenging and risky with each subsequent operation due to scar tissue formation. Similarly, while SRS can be repeated, there are dose constraints to the brainstem that limit this option, and the effectiveness of repeat SRS is often lower than that of the initial procedure [22]. The ability to perform RFR multiple times with a consistent safety profile and a high likelihood of renewed short-term efficacy makes it an exceptionally flexible and durable long-term management strategy for a chronic and recurrent pain condition.

Limitations

This systematic review has several limitations that must be acknowledged. First, the exclusive inclusion of retrospective studies introduces inherent risks of bias, including selection bias, information bias, and confounding. While our risk of bias assessment found six studies to be of low concern, two studies had serious limitations, which may affect the robustness of the pooled findings. Second, there was significant clinical and methodological heterogeneity among the included studies, particularly in terms of patient demographics, specific RFR techniques and parameters, definitions of pain relief and success, and duration of follow-up. This heterogeneity precluded a meaningful meta-analysis and necessitated a cautious, qualitative interpretation of the results. Third, the sample sizes in many studies were relatively small, which limits the statistical power to detect less common adverse events or to perform meaningful subgroup analyses. Finally, the absence of standardized, patient-reported outcome measures across studies means that the impact of treatment on quality of life, patient satisfaction, and the subjective burden of sensory complications is not fully captured, representing a significant gap in the current evidence base.

## Conclusions

RFR is a highly effective intervention for providing rapid and substantial short-term pain relief in patients with MS suffering from TN. Its role is particularly vital for managing acute, severe pain episodes. However, the benefits are often transient, with a high rate of long-term recurrence necessitating a management strategy that anticipates and plans for repeat procedures. The safety profile, characterized by common but often acceptable sensory side effects, and the demonstrated repeatability of the procedure make RFR a cornerstone in the long-term, multimodal management of MS-TN. It occupies a specific niche, offering a balance of high initial efficacy and procedural flexibility that is distinct from other surgical options. Future research should prioritize prospective, randomized studies comparing RFR directly with other percutaneous techniques and should incorporate standardized patient-reported outcomes to better evaluate the true impact of this treatment on the lives of patients with this challenging condition.

## Appendices

**Table 4:** Detailed search string for each database

Database	Search string
PubMed	("trigeminal neuralgia"[Mesh] OR "trigeminal neuralgia"[tiab] OR "tic douloureux"[tiab]) AND ("multiple sclerosis"[Mesh] OR "multiple sclerosis"[tiab] OR "MS"[tiab]) AND ("radiofrequency rhizotomy"[tiab] OR "radiofrequency thermocoagulation"[tiab] OR "radiofrequency ablation"[tiab] OR "radiofrequency lesioning"[tiab]) AND ("pain relief"[tiab] OR "pain control"[tiab] OR "pain outcome"[tiab])
Scopus	(TITLE-ABS-KEY("trigeminal neuralgia" OR "tic douloureux") AND TITLE-ABS-KEY("multiple sclerosis" OR "MS") AND TITLE-ABS-KEY("radiofrequency rhizotomy" OR "radiofrequency thermocoagulation" OR "radiofrequency ablation" OR "radiofrequency lesioning") AND TITLE-ABS-KEY("pain relief" OR "pain control" OR "pain outcome"))
Web of Science	TS=("trigeminal neuralgia" OR "tic douloureux") AND TS=("multiple sclerosis" OR "MS") AND TS=("radiofrequency rhizotomy" OR "radiofrequency thermocoagulation" OR "radiofrequency ablation" OR "radiofrequency lesioning") AND TS=("pain relief" OR "pain control" OR "pain outcome")
Embase	('trigeminal neuralgia'/exp OR 'trigeminal neuralgia':ti,ab OR 'tic douloureux':ti,ab) AND ('multiple sclerosis'/exp OR 'multiple sclerosis':ti,ab OR 'MS':ti,ab) AND ('radiofrequency rhizotomy':ti,ab OR 'radiofrequency thermocoagulation':ti,ab OR 'radiofrequency ablation':ti,ab OR 'radiofrequency lesioning':ti,ab) AND ('pain relief':ti,ab OR 'pain control':ti,ab OR 'pain outcome':ti,ab)
